# Dramatic Reductions in Cigarette Smoking Prevalence among High School Youth from 1991 to 2022 Unlikely to Have Been Undermined by E-Cigarettes

**DOI:** 10.3390/ijerph20196866

**Published:** 2023-09-30

**Authors:** Cristine D. Delnevo, Andrea C. Villanti

**Affiliations:** 1 Rutgers Institute for Nicotine & Tobacco Studies, New Brunswick, NJ 08901, USA; andrea.villanti@rutgers.edu; 2 Rutgers School of Public Health, Piscataway, NJ 08854, USA

**Keywords:** youth smoking, tobacco control

## Abstract

There is concern that youth e-cigarette use could lead youth to initiate cigarette smoking. This study identifies epochs of cigarette smoking among U.S. high school students in three commonly utilized national school-based surveys over three decades without a priori assumptions. We examined trends in ever and current cigarette smoking among high school youth from 1991 to 2022 in three datasets: Monitoring the Future (MTF), the National Youth Risk Behavior Survey (NYRBS) and the National Youth Tobacco Survey (NYTS) via Joinpoint regression. High stable rates of ever smoking were noted from 1991 to 1999 (NYRBS and MTF) and then significantly declined from 1999 to 2013; declines accelerated through to 2022. In the NYTS, ever cigarette smoking significantly declined from 1999 to 2018 and then declines accelerated to 2022. Current cigarette smoking reached its peak in 1997, and then significantly declined from 1997 to 2013 in the NYRBS and MTF and similarly in the NYTS from 1999 to 2018. Declines in current smoking then accelerated in all surveys through to 2022. These findings suggest dramatic successes in reducing youth smoking since the late 1990s, with more rapid declines in prevalence in the past decade.

## 1. Introduction

Cigarettes are overwhelmingly responsible for the bulk of tobacco-related deaths and disease in the United States and accordingly their use has been a public health priority since the first Surgeon General’s Report on Smoking in 1964 [1]. Historically, cigarette smoking has typically begun during adolescence, and 9 out of 10 adults who regularly smoke cigarettes tried their first cigarette by the age of 18 [1]. In 1980, the US Department of Health and Human Services issued the first “Healthy People” public health targets which has included a goal on reducing cigarette smoking in youth since then, with recent goals of reaching 16% prevalence by 2010 and 2020, and 3.4% by 2030 for high school students.

While cigarette companies have targeted youth for decades as “replacement smokers”, in the late 1980s they initiated aggressive youth-targeted marketing (e.g., “Joe Camel”) which successfully increased cigarette smoking and market share among young people [2,3]. The aggressive youth-targeted cigarette marketing prompted the 1998 Master Settlement Agreement (MSA) between 46 states and the tobacco industry, which began a period of considerable changes in the cigarette policy marketplace. First, the MSA limited cigarette marketing to reduce youth exposure and funded large-scale state and national efforts to reduce youth smoking [1]. The MSA’s intense focus on cigarettes facilitated and coincided with the passage of other policies, such as smoke-free air laws and cigarette excise taxes. The evidence base is well established that higher cigarette prices lead to a reduction in youth smoking prevalence [4]. Notably, from 2000 to 2006 the average state cigarette excise tax doubled, and in 2009, Congress also increased the federal tax on cigarettes (USD 0.39 to USD 1.01). The 2009 passage of the Tobacco Control Act gave the FDA regulatory authority over cigarettes and smokeless products, followed by other products, including e-cigarettes, in 2016. Moreover, the Tobacco Control Act reinforced a focus on youth prevention, such as the FDA’s Real Cost Campaign which launched in 2014 [5].

The emergence of e-cigarettes and their uptake among youth, which has been measured in national studies since 2011, prompted concern that e-cigarettes could lead youth to begin cigarette smoking and erase gains made in reducing youth smoking prevalence. Indeed, one analysis of the National Youth Tobacco Survey (NYTS) suggests that the rate of decline in past 30-day cigarette smoking among youth has slowed with the onset of e-cigarettes and this provides evidence of a possible population “gateway effect” from e-cigarettes to cigarettes [6]. However, this particular study used a limited set of years of NYTS data and defined the “e-cigarette era” as beginning in 2014. Moreover, to date, studies on youth cigarette smoking have focused on a single dataset and/or a limited set of years [6,7,8,9,10], obscuring the ability to compare trends in the 30-year surveillance history. This study identifies epochs of cigarette smoking among U.S. high school students in three commonly utilized national school-based surveys: Monitoring the Future (MTF), the National Youth Risk Behavior Survey (NYRBS) and the National Youth Tobacco Survey (NYTS) [1]. Rather than make a priori assumptions about time periods to identify shifts in trends, we employ Joinpoint regression to identify statistically significant inflection points.

## 2. Materials and Methods

Details on the Monitoring the Future study (MTF), National Youth Risk Behavior Survey (NYRBS) and National Youth Tobacco Survey (NYTS) can be found elsewhere [1]. All three school-based surveys evolved over time from paper and pencil administration to computer/tablet administration. All three surveys collect data from middle- and high-school-aged students; for the purpose of our analyses, we focus on estimates for 12th graders (MTF) and high school students (NYRBS and NYTS).

The Monitoring the Future (MTF) study, conducted by the University of Michigan, employs a multistage sampling design to obtain nationally representative samples of secondary school students (i.e., 8th-, 10th-, and 12th-grade students) from the 48 contiguous states [1,11]. Data have been collected annually from 12th-grade students since 1975 and from 8th-and 10th-grade students since 1991. The annual sample size for 12th-grade students was roughly 15,000 until 2020, when it dropped, and was 9500 in 2022. The MTF survey focuses on alcohol and drug use, including tobacco, as well as related attitudes.

Coordinated by the Centers for Disease Control and Prevention (CDC), the National Youth Risk Behavior Survey (NYRBS) has been monitoring health risk behaviors among high school students, biennially, since 1991 [1,12]. In brief, the national survey utilizes a three-stage cluster sample design to obtain a nationally representative sample of students in grades 9 through 12. The NYRBS survey assesses a variety of health risk behaviors, which include tobacco product use.

The CDC developed the Youth Tobacco Surveillance System in 1998 to provide data to support the design, implementation, and evaluation of tobacco control programs at the state and national levels [1,13]. Historically, the National Youth Tobacco Survey (NYTS) and NYRBS use identical sampling methodologies and the same wording for shared survey items. Like the NYRBS, the NYTS High School sample seeks to obtain a nationally representative sample of students in grades 9 through 12. The NYTS began biennially but as of 2011 collects data annually. The NYTS survey focuses solely on tobacco product use.

Our measures of ever and current cigarette smoking are consistent with that used in Surgeon General Reports [1]. Ever smoking is defined as any experimentation with cigarettes; MTF enquires about smoking cigarettes at least “once or twice” and NYRBS and NYTS ask if one has “ever tried cigarette smoking, even one or two puffs” [1]. Survey items for current smoking vary; MTF asks “How frequently have you smoked cigarettes during the past 30 days?” whereas the YRBS and YRBS ask “During the past 30 days, on how many days did you smoke cigarettes?” While the survey questions vary, all three surveys operationally define current smoking as having smoked at all in the 30 days preceding the survey [1].

Trends in ever and current cigarette smoking from 1991 to 2022 in all three surveys were assessed via Joinpoint regression using Joinpoint version 4.9.0 (National Cancer Institute), a segmented regression analysis application. Joinpoint regression analysis is utilized to study varying trends over time in order to identify the time point(s) in which the trend significantly changes. These “joinpoints” are considered inflection points. We present the best model fit, with each segment described by its short-term trend (annual percentage changes [APCs]). Statistically significant APC changes are noted as those with 95% CIs that did not cross 0 (2-sided α  <  0.05). Tests of significance use a Monte Carlo Permutation method. 

## 3. Results

**Figure 1:**
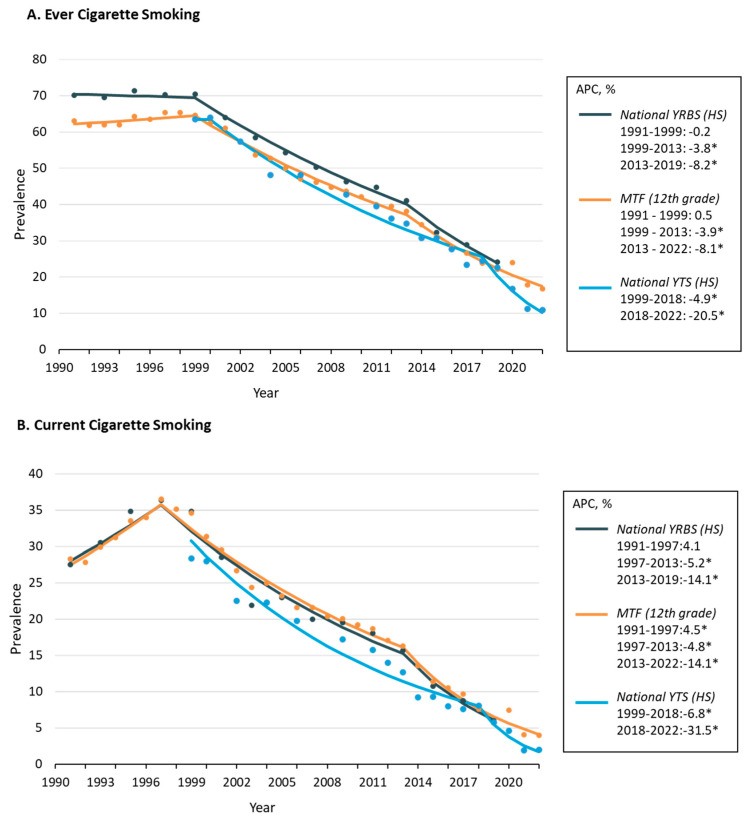
Cigarette smoking trends over time among US high school youth in three national surveys, 1991–2022. Note: APC indicates annual percentage change; * *p*  ≤  0.05.

In 1991, roughly two out of three high school students reported ever smoking cigarettes. Specifically, in 1991, 63.1% of 12th-grade students in the MTF and 70.1% of high school students in the YRBS reported having ever smoked cigarettes. These high rates of ever cigarette smoking were sustained from 1991 to 1999 in both the NYRBS and MTF (Figure 1A), then after nearly a decade of stagnation, ever cigarette smoking significantly declined from 1999 to 2013 in the NYRBS (APC, −3.8% [95% CI, −3.4–4.3%]) and MTF (APC, −3.9% [95% CI, −3.3–4.4%]). Significant declines in ever smoking then accelerated through to 2022, with similar APCs (−8.1% and −8.2%). In the NYTS, ever cigarette smoking significantly declined from 1999 to 2018 (APC, −4.9% [95% CI, −4.2–5.6%]) and then rapidly declined from 2018 to 2022 (APC, −20.5% [95% CI, −14.5–26.0%]). The ever cigarette smoking rate among high school students in the 2022 NYTS was 10.9% and 16.8% among 12th-grade students in the MTF.

As noted in Figure 1B, current cigarette smoking among high school youth reached its peak in 1997, with MTF demonstrating significant increases from 1991 to 1997 (APC, 4.5 [95% CI, 0.2–8.9%]) and the NYRBS showing similar increases (APC, 4.1 [95% CI, −2.8–11.5%]). In 1997, 36.5% of 12th-grade students in MTF and 36.4% of high school students in the NYRBS reported currently smoking cigarettes in the past 30 days. Current cigarette smoking then significantly declined from 1997 to 2013 in the NYRBS (APC, −5.2 [95% CI, −3.4–6.9%]) and MTF (APC, −4.8 [95% CI, −3.9–5.8%]) and similarly in the NYTS from 1999 to 2018 (APC, −6.8 [95% CI, −5.4–8.2%]). Significant declines in current smoking then accelerated in all surveys through to 2022, with the APCs ranging from −14.1 to −31.5. The current cigarette smoking rate among high school students in the 2022 NYTS was 2.0% and 4.0% among 12th-grade students in the MTF.

## 4. Discussion

Between 1991 and 2022, cigarette use among U.S. high school students tracked closely with key periods in the history of tobacco control [1,14] across estimates from three national school-based surveys. Current cigarette smoking reached an all-time high in 1997 following an era of youth-targeted tobacco marketing which culminated in the 1998 MSA. Reductions in current cigarette smoking occurred during the first decade of the MSA, with steeper reductions beginning in 2013, coinciding with the beginning of FDA’s Real Cost Campaign aimed at preventing youth cigarette smoking initiation [5] and the relaunch of the national truth campaign in 2014 [15]. These findings suggest dramatic successes in reducing adolescent smoking prevalence since the late 1990s. However, in contrast to previous analyses of the NYTS which suggest that the declines in cigarette smoking have slowed during the “e-cigarette era’’ [6,8] and as such, provide population-level evidence for the “gateway effect” [6], we found that the most rapid declines in cigarette prevalence have occurred in the past decade, when e-cigarettes emerged as a popular product among youth.

E-cigarette use among high school youth has been tracked in the NYTS since 2011. Past 30-day use remained low (below 5%) until 2014, when it was estimated to have increased three-fold to 13.4% [16]. However, changes in NYTS measures and survey design occurred between 2013 and 2014, which CDC recognizes as potentially limiting the comparability of estimates across years [17]. Indeed, our own empirical analysis of the changes in e-cigarette survey questions used in the NYTS from 2013 to 2014 found that estimates prior to 2014 were likely underestimated, and while there was likely an increase in e-cigarette use from 2013 to 2014, it was unlikely to be a three-fold increase [18]. E-cigarette use among high school youth then decreased from 2015 to 2017, and then increased sharply again in 2018 to 20.8 and reached an all-time high in 2019 of 27.5% [16,17,19]. Tobacco control policies and regulations were quickly initiated at the state and federal levels and by 2022, past 30-day use of e-cigarettes among HS students in the NYTS was estimated at 14.1% [13].

E-cigarettes are now the most popular product used by high-school-aged young people and their use should not be dismissed. While the high prevalence of use is concerning, so is the fact that youth tobacco use is complex and experimentation with multiple tobacco products is common. There is limited support for a gateway theory among youth in the US, which argues that e-cigarettes are uniquely responsible for experimentation with cigarettes or other tobacco products. Instead, patterns of youth experimentation with multiple tobacco products and multiple substances are consistent with the common liability theory, which suggests that the propensity to try tobacco products influences patterns of use [20]. It is notable that e-cigarettes, as the most prevalent product used by youth, are likely to be the first tobacco product tried; however, longitudinal studies support that trying any non-cigarette tobacco product increases the odds of trying cigarettes [21].

This ecological study is limited in its ability to ascribe causality between contextual drivers (e.g., tax/price, policy/regulation, industry marketing, tobacco control programs) and changes in adolescent cigarette use. However, a strength of this study is that we did not make a priori assumptions about the magnitude of trend shifts based on contextual drivers. Notably, others have defined 2014 as a critical time point—marking the beginning of the youth “e-cigarette era” due to the large increases noted in the NYTS from 2013 to 2014 in past 30-day e-cigarette use [6,8]. However, as noted previously, methodological changes in the NYTS likely resulted in an overstatement of the magnitude of the increase in e-cigarette use estimates between 2013 and 2014 [18]. Additionally, ever and past 30-day current use are crude measures of cigarette smoking behavior. Measure of ever and current cigarette smoking prevalence can encompass a wide range of behaviors from first time experimentation to regular, social smoking to daily, long-term sustained use. As such, these measures, while traditional public health surveillance measures, do not account for smoking frequency or intensity. Importantly, smoking frequency and intensity are important considerations in the context of increased tobacco-related harms. Importantly, the published literature clearly points to the fact that in addition to decreases in prevalence, smoking frequency and intensity have also declined considerably in the last two decades [7,22]. Indeed, at the peak of the youth current cigarette smoking prevalence in 1997, the percent of high school youth who smoked daily was 12.2%, which has declined considerably over two decades to 2% [7]. Importantly, an emerging body of evidence suggesting that initiation of cigarette smoking is shifting from adolescence to young adulthood. However, the prevalence of cigarette smoking has also dramatically declined among young adults aged 18–24 from 26.8% in 2000 to 7.4% in 2020 [23].

## 5. Conclusions

Healthy People’s 2030 goal for youth cigarette smoking, which uses the NYTS as its benchmark, has already been achieved and exceeded, years ahead of schedule. Concerns about a potential rise in adolescent cigarette use following the introduction of e-cigarettes to the U.S. market in the early 2010s are not supported by the data. In fact, the emergence of e-cigarettes has coincided with the most rapid declines in cigarette use over the past thirty years. It is important to recognize the possibility that had e-cigarettes not been available, changes in cigarette smoking prevalence among youth may have been different; this includes slower or faster declines. However, given the lack of a counterfactual, it is not possible to empirically evaluate this.

Nonetheless, public health targets should expand efforts to reduce combustible tobacco use in young adults, given important shifts in initiation and progression in this age group. Novel tobacco control efforts have targeted sales bans based on year of birth to reduce youth use (tobacco-free generations) and been met with industry opposition, as have flavored tobacco bans, now implemented in five U.S. states and over 360 localities. A new epoch of cigarette use will likely emerge following regulatory policies that change the nature of cigarette products themselves—removing menthol and reducing nicotine to non-addictive levels. The extent to which these policies experience the successes of earlier epochs will depend largely on the compliance of cigarette companies and continued pressure from public health and tobacco control efforts to reduce the harms associated with combusted tobacco use.

## Data Availability

The data used in this paper are publicly available.
